# Social network type contributes to purpose in life among older people, mediated by social support

**DOI:** 10.1007/s10433-024-00799-w

**Published:** 2024-01-17

**Authors:** Maryam Bakhshandeh Bavarsad, Christine Stephens

**Affiliations:** School of Psychology, Massey University, Private Bag 11-222, Palmerston North, New Zealand

**Keywords:** Structural equation models, Quality of life, Successful ageing

## Abstract

A sense of Purpose in Life is an important aspect of ageing well which is related to older adult’s social relationships. Social network types and the different sources of support they provide are theorized here as a pathway to maintaining a sense of purpose as we age. The study utilized a population sample from the 2016 and 2020 NZ Health, Work, and Retirement (NZHWR) longitudinal survey waves; *N* = 2869 (mean age of 65.82 years (*SD* = 6.40). A structural equation model investigated the relationship between Social Network Type and Purpose in Life and the mediating role of Social Support. The final model had a good fit to the data and explained 33.7% of the variance in Purpose in Life. Private, Self-Contained and Family Dependent network types (2016) were negatively related to Purpose in Life 4 years later (2020). Support from sense of Attachment, Reassurance of Worth, Reliable Alliance and Social Integration played a mediating role between Social Network type and Purpose in Life. These findings highlight the importance of social networks in maintaining a sense of purpose among older adults and highlight pathways for the types of networks, and kinds of social support they offer, which contribute to a sense of purpose in life. They underscore the importance of social support for the well-being of older adults and highlight the need to consider the quality and type of social networks and support they provide, when designing interventions to enhance well-being.

## Background and objectives

A sense of Purpose in Life is receiving increasing attention in gerontological research as an important aspect of ageing well. According to McKnight and Kashdan (2009), purpose in life is a fundamental, self-organizing life objective that arranges and inspires goals, regulates behaviours, and offers a sense of meaning. Three key components of purpose in life among older adults have been identified: having a stable and enduring long-term goal; being motivated to pursue a meaningful goal that extends beyond oneself; and making consistent progress towards achieving the goal (AshaRani et al. 2022). Studies have shown that older adults with a sense of purpose have better cognitive function (Kim et al. 2019), better physical function, and a lower risk of developing sleep problems, stroke, and unhealthy BMI (Kim et al. 2020, 2013). Steptoe and Fancourt (2019) found that the sense that one is living a worthwhile and meaningful life predicts positive changes in social, economic, health, and behavioural outcomes over a four-year period, independent of baseline levels. Given the significant influence of purpose in life on health and well-being outcomes, there is growing interest among healthcare providers and scientists in investigating this phenomenon, particularly among older people who may have decreasing levels of purpose in life as they age (Pinquart 2002). To effectively promote purpose in life as an intervention to improve well-being, it is important to understand the factors that influence sense of purpose and how they do so. In this paper we explore social networks and the social support they provide as important contributors to a sense of purpose in life among older people.

Social relationships are one of the determinants of purpose in life (AshaRani et al. 2022), with sustainable social ties being a common means of deriving a sense of purpose among older adults (Wong 1998). Pinquart (2002) used meta-analysis to demonstrate a robust correlation between purpose in life and the quality of older people’s social contacts as well as their social integration. Steptoe and Fancourt (2019) also reported that a sense of purpose in life is associated with stronger personal relationships, broader social engagement and less loneliness. A closer look at the nature of these social relationships (Pfund et al. 2022) showed that the composition of social networks, the frequency of interactions, and the quality of relationships within social networks can influence older adults' sense of purpose in life. It was shown that older adults who have gratifying social interactions tend to feel more purposeful.

Social networks among older adults are defined as the web of social relationships, including family, friends, and acquaintances (Doubova Dubova et al. 2010), which are a fundamental aspect of life satisfaction, mental health, and overall well-being (Litwin and Shiovitz-Ezra 2010). Only a few studies have explored the relationship between social networks and purpose in life and some of these have only examined specific aspects, such as the size of the network or the presence of social interaction (Lee and Martin 2023; Pfund et al. 2022). The concept of social network type allows for a comprehensive understanding of the structure, function, and quality of social networks among older adults (Cheng et al. 2014; Litwin 1998). Studies have demonstrated that diverse social network types, which include relationships with individuals from various backgrounds and high social interaction and shared activities, are associated with better mental and physical health. Conversely, social network types that are private or isolated are associated with worse mental and physical health (Park et al. 2019; Sohn et al. 2017). As individuals age, their social networks often undergo significant changes, including shifts in size, composition, and purpose. Older adults commonly experience a reduction in the size and diversity of their social networks due to factors such as retirement, relocation, and the loss of close friends and family members (Cornwell et al. 2008) and this may be related to the decline in purpose in life (Pinquart 2002). The demonstrated link between social networks and purpose in life provides a focus for investigation into social networks as an important contributor to purpose in life and we propose a pathway from social networks, through social support, to purpose in life (which subsequently influences health).

The studies of purpose in life in the gerontological literature to date largely provide correlational evidence for links between purpose in life and a range of variables, of which social relationships are a consistent feature, pointing to their importance. However, there is very little theorizing regarding pathways to purpose in life, or how social networks are related to purpose in life. Social networks may offer different levels of social support (Stephens et al. 2011; Szabó et al. 2023; Wenger 1997) and Weston et al. (2021) have shown that receiving more social support is related to having a purposeful life. Social support has long been accepted as an important contributor to health (Callaghan and Morrissey 1993) and this has been explained in terms of social support’s mitigation of the negative effects of stress, promotion of adaptive coping strategies, and improvement of psychological and physical well-being (Berkman et al. 2000). Berkman and colleagues also explain how the structural aspects of social networks such as size and composition, provide access to different types of social support, while functional aspects, such as emotional closeness and perceived supportiveness, can influence the quality and effectiveness of social support.

Social support can be provided in various ways, including emotional support (e.g., listening and empathy), informational support (e.g., advice and guidance), and instrumental support (e.g., tangible assistance). Social provisions theory (Weiss 1974) proposes a more nuanced categorization of the supportive provisions of social relationships which has proven fruitful in assessing the relationship between support and well-being (Yarcheski et al. 2001). According to social provisions theory, the provision of Attachment refers to emotional closeness with others, while Social Integration measures an individual's perceived involvement with people who share their interests and beliefs, and Reassurance of Worth assesses whether individuals feel valued by others (Weiss 1974). These aspects of emotional support provide a promising approach to understanding which perceptions of social support may contribute to a sense of purpose in later life. In addition, social provisions theory includes perceptions of tangible support (Reliable Alliance evaluates whether they can depend on others for help) and informational support (Guidance measures the degree to which individuals receive advice or information from others) while Opportunity for Nurturance assesses whether individuals have opportunities to provide care and support to others.

We aimed to explore the relationship between social network type and purpose in life among older adults, while examining the mediating role of different aspects of social support in this relationship. The research questions are as follows:

1. Which social network types are related to purpose in life over time?
2. Which types of social support mediate any relationships between social network types and purpose in life over time.

## Research design and methods

### Study design and research participants

This prospective study utilized data from the 2016 and 2020 Health, Work, and Retirement (HWR) surveys, a longitudinal study which has been conducted biennially since 2006 to observe and explain the factors associated with health, retirement, and ageing well in the older population of New Zealand. All independent variables were assessed in 2016 (T1), and the dependent variable (Purpose in Life) was assessed in 2020 (T2).

The research participants were randomly selected from individuals aged 55 and above registered on the New Zealand electoral roll (an enrolment rate of 98% of eligible voters). To ensure adequate representation of the indigenous people of New Zealand, individuals of Māori descent were oversampled. Participants received a 24-page postal questionnaire with introductory letter, information sheet, and a return-addressed reply-paid envelope. The return of a full or partially completed survey form was considered as implied consent to participate in the survey.

The present sample included 2869 participants with a mean age of 65.82 years (SD: 6.40). The majority were female (55.9%) and most reported being married or in a de facto relationship (75.0%) and had completed post-secondary or trade education (34.7%). Most reported ‘good’ standard of living (66.2%), with only a small proportion reporting ‘hardship’ (9.0%). In terms of ethnicity, most participants identified as New Zealand Europeans (66.4%) while 24.9% identified as Māori (see Table 1).

**Table 1:** Characteristics of the study population (*n* = 2869)

Variables	Level	Frequency	Per cent
Gender	Male	1262	44
	Female	1605	55.9
	Gender diverse	2	0.1
Marital Status	Married	2151	75
	Not married	718	25
Education Status	No qualifications	583	20.3
	Secondary school	649	22.6
	Post-secondary/trade	996	34.7
	Tertiary	641	22.3
Socioeconomic Status	Hardship	258	9
	Comfortable	711	24.8
	Good	1900	66.2
Ethnicity	New Zealand European	1904	66.4
	Maori	715	24.9
	Other	250	8.7
Social Network Type	Family Dependent	359	16.6
	Locally Integrated	802	37.2
	Local Self-Contained	687	31.8
	Wider Community	179	8.3
	Private	131	6.1

### Measures

#### Demographic characteristics

Ethnicity, age, gender, marital status, and education status were assessed with closed-response questions. Ethnicity was assessed using a self-reported list of options that reflected the diverse ethnic groups in New Zealand, including Māori, New Zealand Europeans, and other ethnicities. To include ethnicity as an independent variable in the SEM, we created dummy variables for Māori (Māori ethnicity = 1; non-Māori = 0) and NZ European (NZ European = 1; non-NZ European = 0). Age was measured in years. Gender was assessed using a self-reported list of options that included male, female, and diverse. To include gender as a predictor variable in the SEM, we created a dummy variable (male = 1; female or diverse = 0). Responses to the range of marital status were dichotomized as partnered (1) and not partnered (0).

#### Socioeconomic status (SES)

SES was evaluated as a composite variable including economic living standards and level of educational attainment. The Economic Living Standards Index-Short Form (ELSI-SF) is a 25-item questionnaire developed in New Zealand to assess the level of the economic well-being (Jensen et al. 2005) by evaluating ownership restrictions, social participation restrictions, economic behaviours, and self-rated indicators of standard of living. ELSI-SF is scored by combining the scores on each item to form a continuous variable ranging from 0 to 31, with higher scores indicating higher economic living standards. The scale showed a Cronbach's alpha coefficient of 0.88 in the current sample. For descriptive purposes, the scores were categorized into three levels: hardship, comfortable, and good. Participants' educational qualifications were categorized into four groups: no qualifications, secondary school, post-secondary/trade, and tertiary.

A composite variable (Ley 1972) combined two dimensions of SES—educational attainment and economic status—into a single category called "SES". Those with no qualification and experiencing economic hardship were categorized as having poor SES (0). The reference category included those who had obtained a secondary school, post-secondary/trade, or tertiary qualification and had a comfortable or good economic living standard (1).

#### Purpose in life

The Life Engagement Test (LET) (Scheier et al. 2006) uses self-report to measure an individual's sense of Purpose in Life. The LET comprises six items that assess an individual's engagement with the world around them, their motivation to pursue goals, and their sense of control over their life. The LET has demonstrated good psychometric properties, including high internal consistency (Cronbach's alpha between 0.72 and 0.87) and test–retest reliability (ranging from 0.61 to 0.76). The Cronbach's alpha coefficient for the current sample was 0.82.

#### Social support

The Social Provisions Scale (SPS) comprises 24 items, each rated on a four-point Likert scale ranging from "strongly disagree" (1) to "strongly agree" (4) with four items in 6 subscales derived from social provisions theory (Weiss 1974), to assess Attachment, Social Integration, Reassurance of Worth, Reliable Alliance, Guidance and Opportunity for Nurturance as forms of social support.

Negative scores are reversed and then summed for each social provision, with scores ranging from 0 to 16. The total score is obtained by summing the six subscale scores, with a possible range of 0 to 96 (Cutrona and Russell 1987). Higher scores are equated to higher social support.

SPS has exhibited robust psychometric properties, with Cronbach's alpha coefficients ranging from 0.67 to 0.76 across the six factors and good test–retest reliability, with coefficients ranging from 0.37 to 0.66 (Cutrona and Russell 1987). The current sample showed a Cronbach's alpha coefficient of 0.91 with subscale alpha coefficients ranging from 0.71 to 0.78.

#### Social network type

The Practitioner Assessment of Network Type (PANT; Wenger 1991) has been utilized to identify distinct social network types. The PANT (adapted to local circumstances) comprises 8 items, assessing distance to nearest relative/child/sibling (3 items on a 6-point scale ranging from ‘in the same building’ to ‘more than 3 h travel’); frequency of interactions with relatives/friends/neighbours (3 items; 5-point scale ranging from daily to less often than monthly), and engagement in community/religious activities (2 items; 3-point scale ranging from never to regularly). The responses to the questions were assessed using the identical algorithm created by Wenger for the identification of social network types (PANT; Wenger 1991). Analysis revealed five distinct social network types: locally integrated (strong connections with local family members, friends, neighbours, and the community); locally self-contained (household-focused lifestyle and distant relationships with kin); wider community-focused (extensive contact with friends, neighbours, and relatives who live at a distance and often involves voluntary community organizations), family-dependent (close connections with local family members but few peripheral friendships and little contact with neighbours), and private (absence of local family, few nearby friends, and minimal community contact or involvement). Dummy variables were created for entry into the SEM model. For example, '1' represented private network type, and '0' indicated the absence of private network type. This approach was applied to each network type. The construct validity of the PANT has been supported in survey research of older adults in Aotearoa New Zealand (Szabo et al. 2018) and the utility of the use of Wenger’s algorithm empirically tested on a New Zealand sample (Stephens et al. 2011).

### Analysis

All statistical analysis was performed using SPSS 26.0 and AMOS29.0 software. The Pearson correlation coefficient test was used to assess the relationship between variables. The normality of variables was checked through estimating the skewness and kurtosis, and all measured variables were screened for outliers.

A total of 86 were excluded due to missing data (nominal variables), resulting in a final sample size of 2869. The missing data amounted to less than 5% of the total sample size, supporting the decision to exclude the missing samples instead of imputing the data (Tabachnick and Fidell 2001). For continuous variables, missing data were addressed through Full information maximum-likelihood (FIML) using AMOS software.

A structural equation model (SEM) was used to investigate the relationship between Social Network Type and Purpose in Life and investigate the mediator role of Social Support. As the normality assumption was met, Maximum Likelihood Estimation was applied to estimate measures. The initial model was adjusted by deleting insignificant paths and using the modification index. The Goodness-of-fit of the hypothesized model was evaluated by goodness-of-fit index (GFI), adjusted GFI, root mean square error of approximation (RMSEA), and standardized root mean square residual (SRMR) and incremental fit indices (i.e., comparative fit index (CFI), normed-fit index (NFI). An acceptable fit value of GFI, AGFI and CFI is greater than 0.90 (Kline 1998). An RMSEA < 0.05 suggests a "close fit," and < 0.08 indicates a reasonable model-data fit. (Browne 1993).

The significance of the mediation effect was examined using the standard error, and 95% confidence intervals calculated from 5000 resamples generated by a bias-corrected bootstrapping method. Variance Accounted For (VAF) provides information about the effect size of the indirect effect on the total effect. VAF was calculated by following formula:

$${\text{VAF}}=\left(\frac{\text{Indirect effect}}{\text{Total effect}}\right)\times 100$$

when the VAF value exceeds 80%, it is considered a case of full mediation, while a VAF value between 20 and 80% signifies partial mediation. Conversely, a VAF value below 20% indicates the absence of mediation (Hair et al. 2021).

## Results

Descriptive statistics showed that social support had a mean score of 79.27 (SD = 9.58), Attachment averaged 13.27 (SD = 2.15), Social Integration 13.17 (SD = 1.95), Opportunity for Nurturance 12.11 (SD = 2.31), Reassurance of Worth 13.07 (SD = 1.88), Reliable Alliance 13.96 (SD = 1.90), and Guidance 13.68 (SD = 2.05). The percentage in each social network type is shown in Table 1.

Bivariate correlations (see Table 2) showed that Purpose in Life was negatively correlated with Local Self-Contained and Private social network types. Purpose in Life was positively associated with all types of social support.

**Table 2:** Pearson’s correlation coefficients between the study variables (*n* = 2869)

	Age	SES	NZ European	Māori	Gender(male)	Not partnered	Attachment	Social integration	Nurturance	Worth	Reliable alliance	Guidance	Family dependent	Locally integrated	Local self-contained	Wider community	Private
Age	1.00																
SES	− 0.04*	1.00															
NZ European	0.04*	− 0.09**	1.00														
Māori	0.00	0.09**	− 0.36**	1.00													
Gender(Male)	0.05**	− 0.03	0.01	0.00	1.00												
Not married	0.08**	0.10**	− 0.08**	0.11**	− 0.17**	1.00											
Attachment	− 0.01	− 0.09**	0.08**	− 0.05**	− 0.10**	− 0.24**	1.00										
Social Integration	0.03	− 0.10**	0.10**	− 0.03	− 0.12**	− 0.10**	0.65**	1.00									
Opportunity for Nurturance	− 0.04*	− 0.06**	0.04*	− 0.03	0.04*	− 0.25**	0.36**	0.33**	1.00								
Reassurance of Worth	− 0.02	− 0.11**	0.09**	− 0.06**	− 0.08**	− 0.09**	0.57**	0.60**	0.29**	1.00							
Reliable Alliance	− 0.01	− 0.09**	0.11**	− 0.05*	− 0.11**	− 0.12**	0.69**	0.67**	0.29**	0.59**	1.00						
Guidance	− 0.01	− 0.09**	0.09**	− 0.06**	− 0.10**	− 0.11**	0.75**	0.66**	0.28**	0.60**	0.76**	1.00					
Family Dependent	− 0.13**	0.02	− 0.09**	0.07**	0.01	0.00	− 0.02	− 0.08**	0.06**	− 0.03	− 0.02	− 0.03	1.00				
Locally Integrated	0.12**	− 0.05**	0.00	0.04*	− 0.09**	0.02	0.12**	0.19**	0.07**	0.08**	0.13**	0.12**	-0.24**	1.00			
Local Self-Contained	− 0.03	0.01	0.06**	− 0.05**	0.09**	− 0.02	− 0.07**	− 0.09**	− 0.08**	− 0.04*	− 0.08**	− 0.07**	− 0.21**	− 0.35**	1.00		
Wider Community	0.08**	0.00	0.01	− 0.06**	− 0.04*	− 0.01	0.01	0.05**	− 0.01	0.04*	0.03	0.02	− 0.10**	− 0.16**	− 0.15**	1.00	
Private	− 0.04	0.07**	0.00	− 0.02	0.04*	0.04	− 0.11**	− 0.14**	− 0.06**	− 0.05***	− 0.08**	− 0.07**	− 0.08**	− 0.14**	− 0.12**	− 0.06**	1.00
Purpose in Life	− 0.05**	− 0.12**	0.05**	− 0.03	− 0.06**	− 0.11**	0.44**	0.43**	0.18**	0.44**	0.41**	0.41**	− 0.03	0.09**	− 0.06**	0.02	− 0.09**

*Correlation is significant at the 0.05 level (2-tailed); **Correlation is significant at the 0.01 level (2-tailed)

**Fig. 1:**
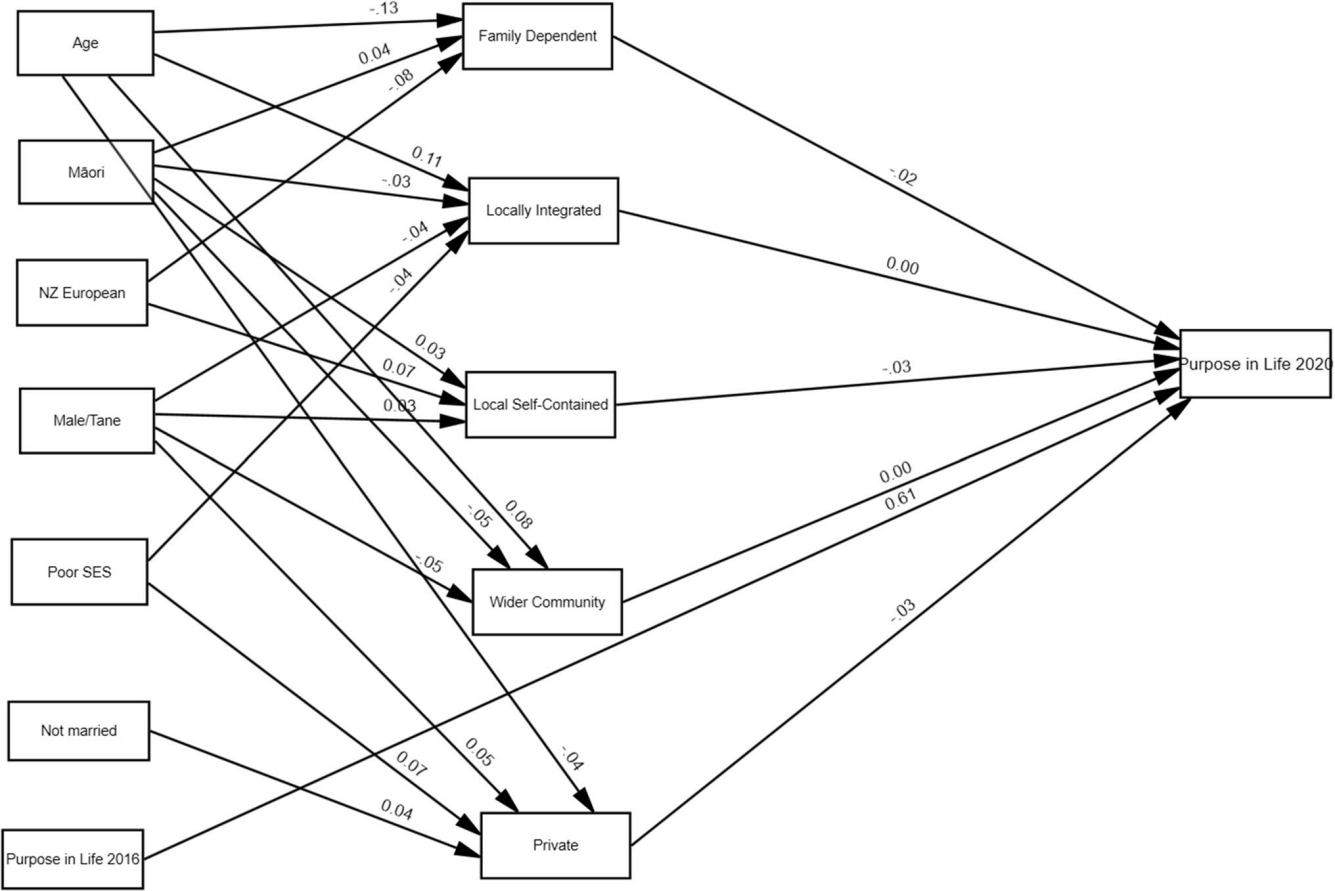
Model illustrating the direct effect of social network types on purpose in life

To answer the first question, the SEM model was depicted without mediators (Fig. 1) to show direct effect between social network types and Purpose in Life. The model had a good fit to the data (GFI = 0.98; AGFI = 0.97; NFI = 0.93; CFI = 0.94; RMSEA = 0.04; SRMR = 0.04). The model showed that there were no direct effects of social network type on Purpose in Life (Table 3).

**Table 3:** The direct relationships between social network types and purpose in life

Dependent variable		Independent variable	*B*	S.E	C.R	*p*	*β*
Purpose in life	←	Family Dependent	− 0.27	0.19	− 1.46	0.15	− 0.025
Purpose in life	←	Locally integrated	− 0.03	0.15	− 0.21	0.84	− 0.004
Purpose in life	←	Local self-contained	− 0.30	0.16	− 1.91	0.06	− 0.035
Purpose in life	←	Wider community focused	− 0.08	0.24	− 0.31	0.76	− 0.005
Purpose in life	←	Private	− 0.44	0.28	− 1.61	0.11	− 0.025

**Fig. 2:**
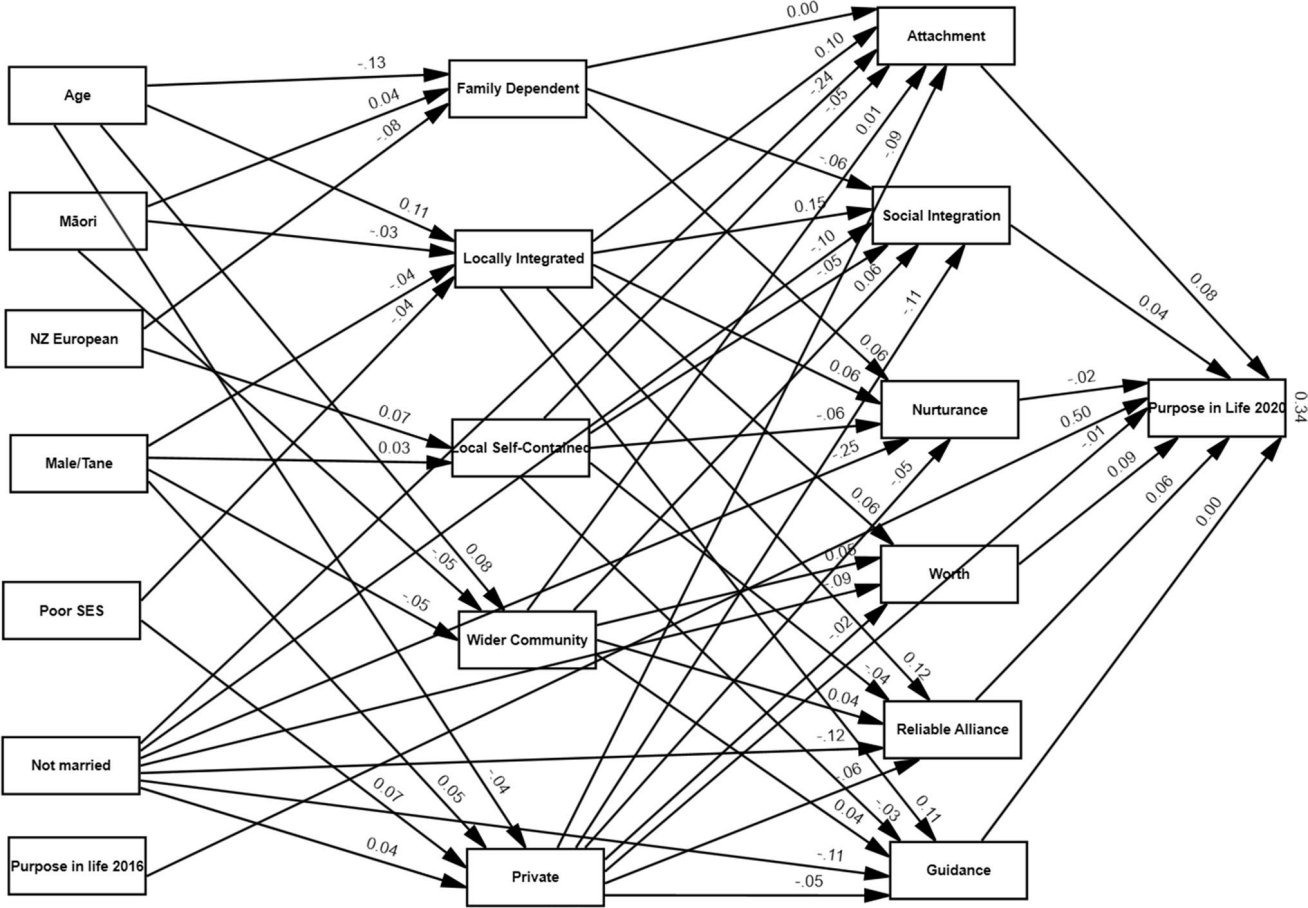
Edited model: the model explained approximately 33.7 per cent of the variance of Purpose in Life. *Note*: The numbers displayed on the paths within the SEM model represent Beta standard coefficients

The model to test the mediating effects of social support in the Social Network and Purpose in Life relationship, with standardized coefficients for direct effects, is shown in Fig. 2. This model was created by removing insignificant paths from the initial model and using modification indexes. The model had a good fit to the data (GFI = 0.96; AGFI = 0.91; NFI = 0.92; CFI = 0.92; RMSEA = 0.07; SRMR = 0.07) and explained 33.7% of the variance in Purpose in Life.

The standardized path coefficients and associated p values for the structural model are shown in Table 4. All forms of social support had a substantial positive impact on Purpose in Life. Reassurance of Worth (*β* = 0.09, *p* < 0.001) and Attachment (*β* = 0.09, *p* < 0.001) had the strongest effects.

**Table 4:** The direct relationships between dependent and independent variables in the structural equation model

Dependent variable	Independent variable	*B*	S.E	C.R	*p*	*β*
Family Dependent	Age	− 0.01	0.00	− 7.47	***	− 0.13
	Māori	0.03	0.01	2.25	0.03	0.04
	New Zealander	− 0.07	0.02	− 4.01	***	− 0.08
Locally integrated	Age	0.01	0.00	6.16	***	0.11
	Gender (Male)	− 0.03	0.01	− 2.60	0.01	− 0.04
	SES	− 0.10	0.04	− 2.26	0.02	− 0.04
	Māori	− 0.03	0.01	− 2.60	0.01	− 0.03
Local self-contained	New Zealander	0.08	0.02	3.88	***	0.07
	Gender (Male)	0.03	0.01	2.43	0.02	0.03
Wider community focused	Age	0.00	0.00	4.21	***	0.08
	Māori	− 0.03	0.01	− 3.81	***	− 0.05
	Gender (Male)	− 0.03	0.01	− 3.81	***	− 0.05
Private	Gender (Male)	0.02	0.01	2.70	0.01	0.05
	SES	0.09	0.02	3.74	***	0.07
	Age	0.00	0.00	− 2.35	0.02	− 0.04
	Not Married	0.02	0.01	2.20	0.03	0.04
Attachment	Family Dependent	− 0.02	0.09	− 0.28	0.78	0.00
	Locally integrated	0.48	0.10	4.90	***	0.10
	Private	− 0.93	0.19	− 4.87	***	− 0.09
	Local self-contained	− 0.24	0.09	− 2.61	0.01	− 0.05
	Wider community focused	0.12	0.16	0.76	0.45	0.01
	Not Married	− 1.19	0.09	− 13.45	***	− 0.24
Social integration	Locally integrated	0.68	0.09	7.55	***	0.16
	Local self-contained	− 0.20	0.08	− 2.57	0.01	− 0.05
	Wider community focused	0.46	0.15	3.14	0.00	0.06
	Private	− 1.01	0.16	− 6.16	***	− 0.12
	Not Married	− 0.45	0.08	− 5.51	***	− 0.10
	Family Dependent	− 0.32	0.11	− 3.00	***	− 0.06
Opportunity for Nurturance	Not Married	− 1.36	0.10	− 14.22	***	− 0.26
	Locally integrated	0.31	0.11	2.92	0.00	0.06
	Private	− 0.50	0.21	− 2.39	0.02	− 0.05
	Local self-contained	− 0.33	0.11	− 3.00	0.00	− 0.06
	Family Dependent	0.38	0.13	2.89	0.00	0.06
Reassurance of Worth	Private	− 0.14	0.15	− 0.92	0.36	− 0.02
	Locally integrated	0.25	0.07	3.51	***	0.07
	Wider community focused	0.33	0.13	2.56	0.01	0.05
	Not Married	− 0.38	0.08	− 4.72	***	− 0.09
Reliable Alliance	Local self-contained	− 0.17	0.08	− 2.17	0.03	− 0.04
	Locally integrated	0.50	0.08	5.89	***	0.12
	Wider community focused	0.36	0.14	2.49	0.01	0.05
	Private	− 0.57	0.17	− 3.33	***	− 0.06
	Not Married	− 0.53	0.08	− 6.55	***	− 0.12
Guidance	Private	− 0.47	0.19	− 2.54	0.01	− 0.05
	Locally integrated	0.50	0.09	5.50	***	0.11
	Not Married	− 0.53	0.09	− 6.08	***	− 0.11
	Wider community focused	0.31	0.16	1.96	0.05	0.04
	Local self-contained	− 0.14	0.08	− 1.62	0.11	− 0.03
Purpose in life	Guidance	0.00	0.05	− 0.06	0.96	0.00
	Attachment	0.14	0.05	3.42	***	0.09
	Social integration	0.08	0.05	2.04	0.04	0.05
	Opportunity for Nurturance	− 0.03	0.03	− 1.18	0.24	− 0.02
	Reliable Alliance	0.11	0.05	2.42	0.02	0.06
	Reassurance of Worth	0.18	0.04	4.54	***	0.09
	Purpose in Life 2016	0.50	0.02	31.28	***	0.50

***p value < 0.001

The model shows that different social network types were related to different forms of social support. Locally Integrated was positively related to all forms of social support. Private Restricted type was negatively related to all forms of social support, except for Reassurance of Worth. Family-Dependent type was positively related to Opportunity for Nurturance while negatively related to Social Integration. Local Self-Contained was negatively related to Attachment, Social Integration, Opportunity for Nurturance, and Reliable Alliance. In contrast, Wider Community-focused was positively related to Social Integration, Reassurance of Worth, Reliable Alliance, and Guidance.

### Indirect effects of social support

The bootstrapping procedure showed that four forms of social support, Social Integration, Attachment, Reassurance of Worth, and Reliable Alliance played a mediator role between Wider Community Focused, Locally Integrated, and Private network types and Purpose in Life (see Table 5). Full mediation (VAF: 88%) was seen for Social Integration as a mediator of the positive effect of Wider Community Focused network type on Purpose in Life (*β* = 0.04, 95% CI [0.00, 0.09). The indirect positive effect of Wider Community Focused network type on Purpose in Life through Reliable Alliance was significant (*β* = 0.04, 95% CI [0.01, 0.11); VAF: 0.89%, indicated full mediation. The indirect positive effect of Locally Integrated network type on Purpose in Life through Reassurance of Worth was significant (*β* = 0.05, 95% CI [0.02, 0.09); VAF: 93% indicated full mediation. The indirect negative effect of Private Network type on Purpose in Life through Social Integration was significant (*β* = − 0.09, 95% CI [− 0.20, − 0.02); VAF: 78%, indicated partial mediation.

**Table 5:** Mediation effect of social provisions on the association between social network type and purpose in life

Regression path			Indirect effect	95% Confidence interval		*p*	Direct effect	Total effect	VAF
				Lower	Upper				
Family Dependent	Social Integration	Purpose in Life	− 0.03	− 0.06	− 0.01	0.04	− 0.025	− 0.055	0.55
Locally Integrated	Attachment	Purpose in Life	0.07	0.03	0.13	0	0.004	0.074	0.95
Locally Integrated	Reassurance of Worth	Purpose in Life	0.05	0.02	0.09	0	0.004	0.054	0.93
Locally Integrated	Reliable Alliance	Purpose in Life	0.06	0.01	0.12	0.02	0.004	0.064	0.94
Local Self-Contained	Social Integration	Purpose in Life	− 0.02	− 0.05	0	0.04	− 0.035	− 0.055	0.36
Local Self-Contained	Reliable Alliance	Purpose in Life	− 0.02	− 0.06	0	0.03	− 0.035	− 0.055	0.36
Wider Community	Social Integration	Purpose in Life	0.04	0	0.09	0.04	0.005	0.045	0.88
Wider Community	Reassurance of Worth	Purpose in Life	0.06	0.02	0.13	0.01	0.005	0.065	0.92
Wider Community	Reliable Alliance	Purpose in Life	0.04	0.01	0.11	0.02	0.005	0.045	0.89
Private	Attachment	Purpose in Life	− 0.13	− 0.27	− 0.05	0	− 0.025	− 0.155	0.84
Private	Social Integration	Purpose in Life	− 0.09	− 0.2	− 0.02	0.04	− 0.025	− 0.115	0.78
Private	Reliable Alliance	Purpose in Life	− 0.07	− 0.17	− 0.01	0.01	− 0.025	− 0.095	0.74

## Discussion

The findings show that the types of social networks within which people conduct their social relations provide forms of social support that, in turn, contribute to a sense of purpose in life over time. While all types of social support were associated with purpose in life, only support from a sense of attachment, reassurance of worth, reliable alliance, and social integration played this mediating role.

To answer question 1, we found that membership in a social network typed did not directly affect sense of purpose in life. Other network types were not directly related to purpose in life. Older people in private restricted networks are at greater risk of low morale, loneliness and depression and psychological illnesses (Wenger 1997) and, according to these findings, to lower sense of purpose in life.

The findings in regard to question 2 suggest that a wider community focused network and a locally integrated network affect purpose in life positively only through the types of support that these network types make available: social integration, reliable alliance, reassurance of worth, and attachment. Conversely, older adults with constrained private networks are more likely to have a lower sense of purpose in life because of the type of social support least likely to be available from this network type: a sense of social integration. The absence of a direct effect between the independent and dependent variables is not a restrictive criterion for assessing the mediation role (e.g., McKinnon et al. 2000; Preacher and Hayes 2004). The significant indirect effect of social network types on sense of purpose in life through aspects of social support suggests that, while a person's sense of purpose in life may not be directly influenced by their social network, it may be indirectly affected by the social support they are more likely to receive from that network. The identification of the indirect effect of the type of social network on purpose in life, through particular aspects of social support is the key contribution of the present study. Below we discuss the ways in which these specific mediation effects may function.

A wider community focused network, including extensive contact with friends, neighbours, and relatives, and community engagement (Wenger 1991) can positively impact a person’s sense of purpose in life by providing social support through shared interests (social integration), the availability of assistance from others in times of need (reliable alliance) and reassurance of worth to others. Membership in such diverse networks increases the likelihood of receiving these forms of social support. The presence of individuals with diverse roles in a social network has previously been shown to increase the likelihood of receiving various forms of social support (Agneessens et al. 2006). A locally integrated network, which has similar qualities plus closer proximity to network members, additionally impacts purpose in life by providing emotional closeness (attachment). It has been shown that individuals in the inner circle of an individual, i.e., those who are highly important to them and whose absence would be unimaginable from their perspective, tend to live close to them and also provide the highest levels of social support (Antonucci and Akiyama 1987).

In contrast, it appears that older adults with small constrained private networks have a lower sense of purpose in life through lower social integration. Private restricted networks have the fewest connections among friends or family and have been shown to be the most likely to be related to loneliness and poor mental health (Stephens et al. 2011; Wenger 1997). Accordingly, members of the private restricted network type were less likely to report support through involvement with others who share their interests and beliefs.

Previous research has shown that different forms of support are provided by family, friends, or neighbours (Agneessens et al. 2006). Companionship is more likely to be found with friends rather than family (Rook and Ituarte 1999) because relationships with friends are based on personal choice, similar characteristics, and lifestyle (Huxhold et al. 2013). This consensus supports the present finding that older adults who are members of wider social networks, with a greater number of diverse connections with friends and neighbours, benefit from a higher level of social integration, attachment, reliable alliance, and reassurance of worth, and, consequently, a greater sense of purpose in life.

It is understandable that these types of emotional and personally confirming support should be related to having a sense of purpose in life. Wong (1998) proposes that it is challenging to attain a meaningful life without social acceptance, based on qualitative work in which individuals identified the attributes of a meaningful life, including being respected, having strong ties with family and friends, and feeling a sense of belonging to a community or group. Pinquart’s (2002) meta-analysis identified that maintaining social relationships in old age and having high-quality relationships play a significant role in maintaining a sense of purpose in life. Our findings provide more details regarding the types of relationships, and the kinds of social support that they offer, which contribute to maintaining that important sense of purpose.

The strengths of this study include a large sample size which includes representation of the indigenous ethnic group of New Zealand and prospective data. The consideration of six types of social support provides a more comprehensive understanding of how social network types affect purpose in life through social support. The use of structural equation modeling has enabled simultaneous modelling of several regression equations and identifying indirect pathways to estimate the contribution of each predictor variable to the covariance structure.

### Implications

These findings highlight the importance of social networks for maintaining a sense of purpose in life among older adults. The findings suggest that different types of social networks can influence purpose in life through the provision of, or lack of, specific types of social support. For example, locally integrated social networks, which provide emotional attachment and promote a sense of self-worth and perceived access to assistance in times of need from others and social integration, can contribute to a meaningful life for older adults. In contrast, a private network that lacks attachment and social integration can be related to reduced sense of purpose in life. While all types of social support are associated with purpose in life, only support from a sense of attachment, reassurance of worth, reliable alliance, and social integration explain the important support that particular network types provide.

This study underscores the importance of social support for the well-being of older adults. The design of interventions to foster an ongoing sense of purpose in life among older people should include consideration of the support provided by different types of identifiable social networks.
